# Supplementary material for: Susceptibility of Glucose Regulation to Social Isolation
Source: bioRxiv. 2025 Dec 22:2025.12.18.695168. Preprint. [Version 1] doi: 10.64898/2025.12.18.695168 (PMC12776040; doi:10.64898/2025.12.18.695168)
Supplement: Supplement 1 [file NIHPP2025.12.18.695168v1-supplement-1.pdf]

bioRxiv preprint doi: https://doi.org/10.64898/2025.12.18.695168; this version posted December 22, 2025. The copyright holder for this preprint (which was not certified by peer review) is the author/funder, who has granted bioRxiv a license to display the preprint in perpetuity. It is made available under aCC-BY 4.0 International license.

Supplementary figures

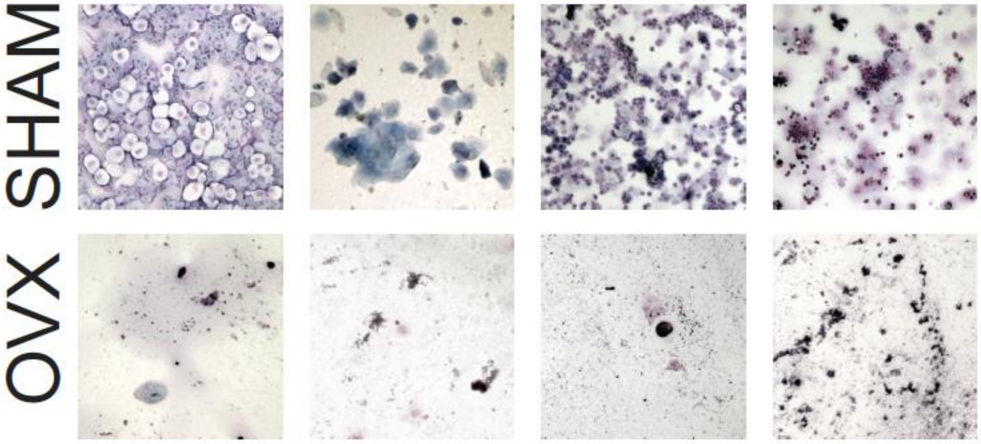

Fig. S1: **Example of vaginal cytology confirming successful ovariectomy in females**
Top, cytology showing different phases of the estrus cycle in sham operated animals. **Bottom**, cytology from OVX females, showing minimal cells.

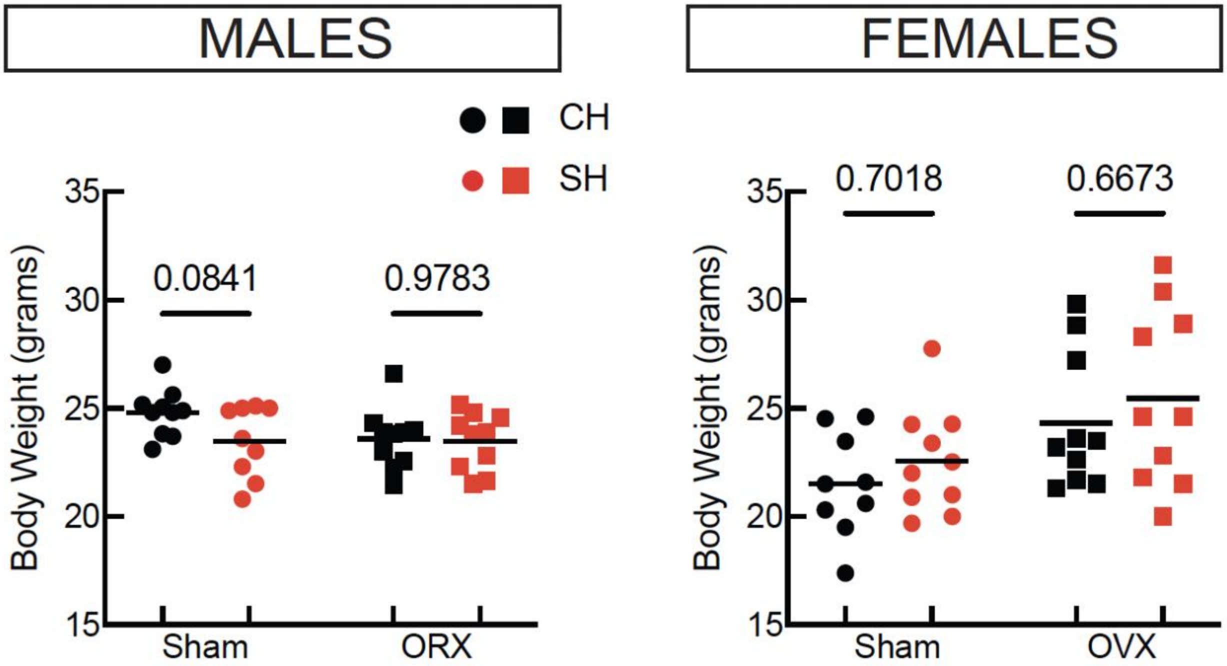

Fig. S2: **The effects of isolation on body weight in intact and gonadectomized mice**
Left, no effect of housing, surgery, or their interaction in male mice (two-way ANOVA, N = 19 sham, 20 ORX). In Sidak's corrected pairwise comparisons there was a trend for lower body weight in isolated compared to co-housed sham animals. **Right**, isolation does not affect body weight in females, but OVX surgery leads to increased body weight (p = 0.007, two-way ANOVA, N = 19 sham, 20 OVX).

bioRxiv preprint doi: https://doi.org/10.64898/2025.12.18.695168; this version posted December 22, 2025. The copyright holder for this preprint (which was not certified by peer review) is the author/funder, who has granted bioRxiv a license to display the preprint in perpetuity. It is made available under aCC-BY 4.0 International license.

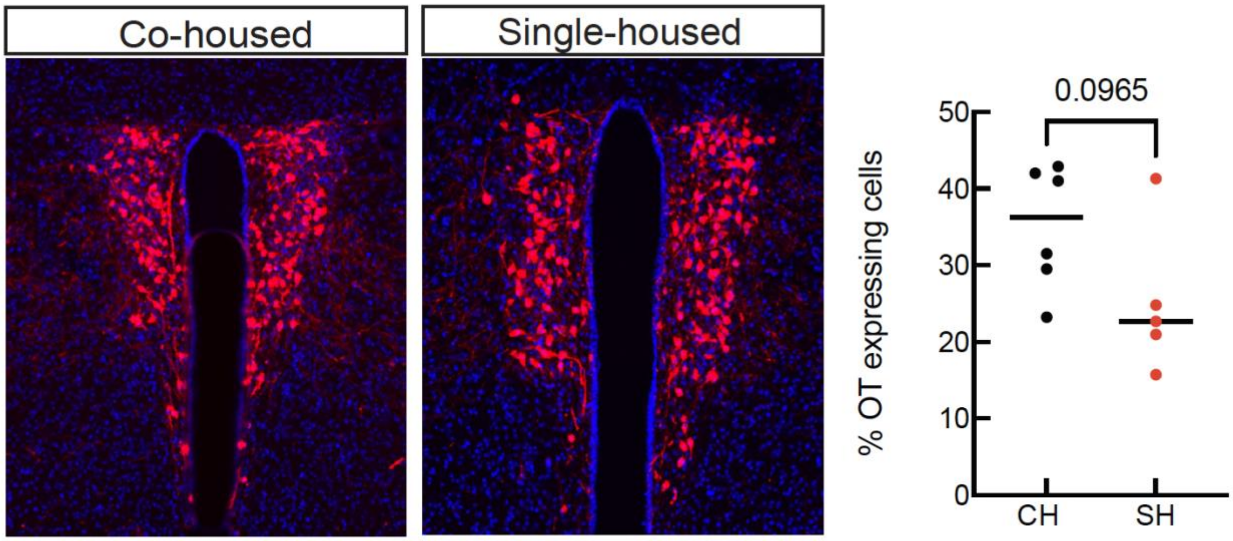

Fig. S3: **Isolation does not significantly change the proportion of oxytocin neurons in PVN**
Left, oxytocin neurons (in red) in PVN. Right, quantification of oxytocin neurons in PVN (unpaired t-test, N = 6 co-housed and 5 single-housed male mice).

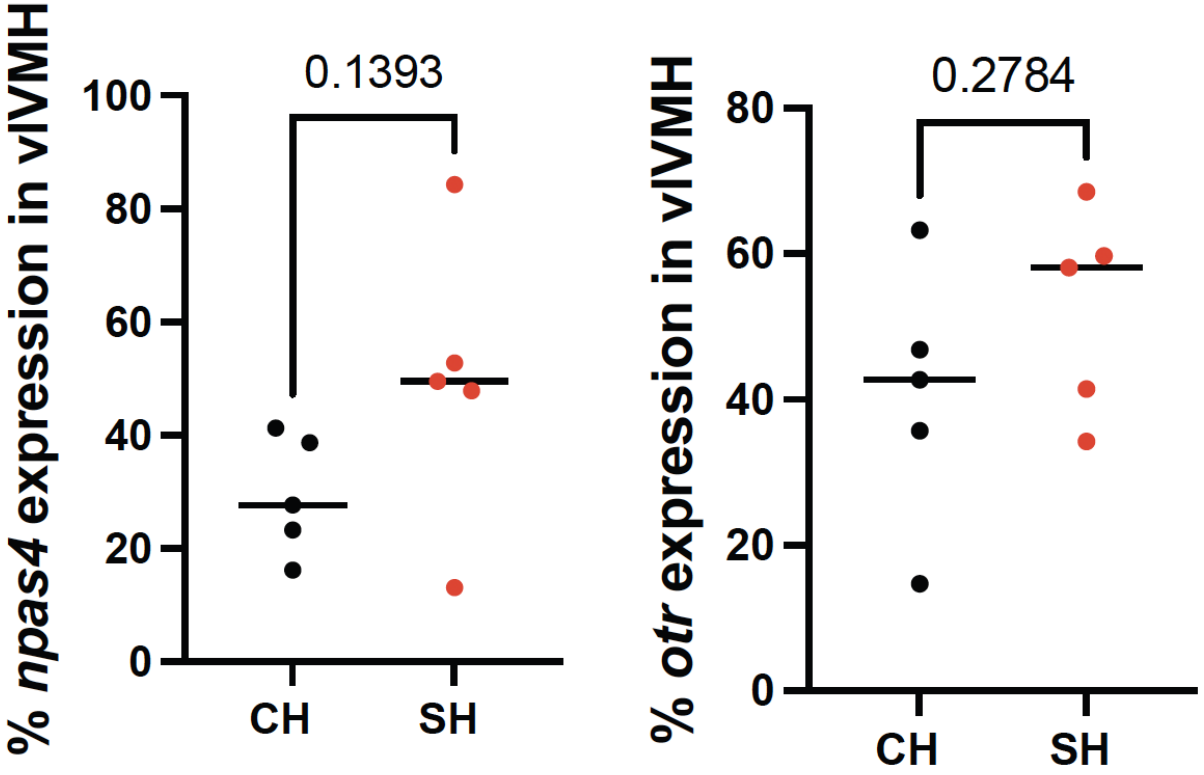

Fig. S4: **Isolation does not significantly change Npas4 and OTR expression in VMH**
Quantification from N = 5 co-housed and 5 single-housed male mice. Unpaired t-test.
